# It’s just that uncertainty that eats away at people: Antarctic expeditioners’ lived experiences of COVID-19

**DOI:** 10.1371/journal.pone.0277676

**Published:** 2022-11-14

**Authors:** Meredith Nash, Elizabeth Leane, Kimberley Norris

**Affiliations:** 1 College of Engineering and Computer Science, Australian National University, Canberra, Australian Capital Territory, Australia; 2 School of Humanities, University of Tasmania, Hobart, Tasmania, Australia; 3 Institute for Marine and Antarctic Studies, University of Tasmania, Hobart, Tasmania, Australia; 4 School of Psychological Sciences, University of Tasmania, Hobart, Tasmania, Australia; University of Exeter, UNITED KINGDOM

## Abstract

With Antarctic expeditioners popularly portrayed in the media during the pandemic as both heroic stalwarts better equipped than any other people to deal with the rigours of isolation and, paradoxically, the only people untouched by the virus, it was all too easy to ignore the actual experiences of those working in the continent. Drawing on the experiences of expeditioners in the Australian Antarctic Program from 2019–21, this article provides a counter to popular media perspective by exploring how COVID-19 protocols–including quarantine and social distancing–affected expeditioners’ individual well-being and their experiences of the social environment. We argue that Antarctic life during COVID-19 has not been as detached from the rest of the world nor as heroic as the popular media has suggested, but nonetheless provides important insights for survival in isolated, confined, and extreme environments (ICE) and non-ICE environments at a time of pandemic.

## Introduction

As the COVID-19 virus spread across the world in 2020, the Australian Antarctic Program (AAP) shared numerous messages of solidarity from wintering expeditioners as well as practical tips on staying well during isolation [1]. The AAP was not alone in this strategy: Antarctic expeditioners at a range of locations were put forward as some of the world’s best coaches on how to survive isolation during a global pandemic. Dramatic headlines reinforced a pre-existing notion of Antarctica as the ultimate testing ground of character [2] and applied it to the social isolation necessary during a pandemic [3]. For instance, British expeditioners working at Port Lockroy on the Antarctic Peninsula were dubbed a ‘plucky team’ of ‘brave adventurers’ [4]. The media coverage activated a pre-existing association of Antarctica with stoic (white male) heroes–sometimes explicitly, as in one *New York Times* article in which US naval officer Richard Byrd, who deliberately isolated himself for several months in the Antarctic interior in the mid-1930s, is held up as “endur[ing] the ultimate in social distancing’ [5]. With the world plunged into unexpected circumstances, the continent of extremes suddenly became the apparent model for survival, reinvoking Antarctica, once again, as a stage for heroic endurance. In this way, Antarctica shifted from being a space analogue which is applicable to a small number of people each year to an analogue for the pandemic, applicable to billions of people worldwide.

Running in ironic parallel with this media discourse of heroism in isolation was the idea of Antarctica as a refuge of normalcy during the global pandemic. The ice continent was repeatedly presented by the media as the “safest place in the world’ [3]–a kind of natural quarantine area. Media articles about isolated expeditioners were frequently accompanied by imagery showing people in pristine natural landscapes, rather than in the more claustrophobic quarters of a station which largely typifies their lived experiences, especially during winter [4]. Expeditioners were quoted expressing their horror at the state of the world outside the sanctuary of Antarctica: one doctor at India’s Bharati station observed, ‘It is beyond my imagination to realise the entire world going out with their masks on’ [4].

Media reports such as these often obscured the mundane reality of living and working in Antarctica during the pandemic. This article fills a critical knowledge gap, as (so far as we are aware) it is the first study to provide empirical data on the lived experiences of expeditioners who arrived in Antarctica before and during the COVID-19 pandemic (2019–21). Here, ‘Antarctica’ is defined to include the Antarctic continent, sub-Antarctic Islands, and the Southern Ocean. In this study, organisational culture provides a compelling frame of reference for understanding how COVID-19 safety protocols re-shaped the social experience of living and working in Antarctica. Interviews with expeditioners provided data about how COVID-19 affected the everyday working practices of expeditioners, the social dynamics on station, and the realities of negotiating COVID-19 hygiene protocols on station and on vessels. Findings are highly applicable to National Antarctic Programs (NAPs)–the government agencies that manage national Antarctic activities–in developing healthy workplace cultures and various countermeasures to improve the performance of individuals and teams during a pandemic. It also provides lessons for other isolated, confined, and extreme (ICE) environments (e.g., the Arctic, Outer Space, undersea, mining). Moreover, the parallels between social distancing, isolation, and confinement in Antarctica and the rest of the world during COVID-19 offer learning opportunities for non-ICE settings.

In the forthcoming sections, we first provide an overview of the relevant multi-disciplinary literature on living and working in ICE environments and organisational culture in Antarctica. We move to a detailed discussion of how NAPs initially responded to the pandemic, with a particular focus on the AAP. Next, we outline the qualitative study methods and results. We explore three key themes, including how study participants learned about COVID-19 in Antarctica; their individual experiences of quarantine and social distancing; and the impact of quarantine and social distancing on social interactions on stations and vessels. To conclude, we discuss how study findings provide a unique lens for understanding the personal and social complexities that lie beyond familiar stereotypes of Antarctic expeditioners and we flag the implications for NAPs in supporting expeditioner well-being.

### Living and working in isolated, confined, and extreme (ICE) environments

Antarctica is routinely described using superlatives–it is the coldest, driest, windiest, and remotest place on earth, making it one of the most difficult places to live and work [6]. Thus, Antarctic expeditioners, like astronauts, are readily positioned as an elite group who can show the rest of the world how to survive in ICE environments [7]. Extreme and unusual environments such as Antarctica are defined along physical, technological, social, and psychological parameters [8]. Researchers have consistently demonstrated that psychological factors, followed by social factors, have the greatest impact on expeditioner health, wellbeing, and performance and as such need to be prioritised by organisations when considering proactive prevention and intervention management approaches [7]. Given parallels between the physical, technological, social, and psychological experiences, Antarctica is an established analogue for long-duration space missions [9]. NAPs carefully choose who will work in Antarctica because expeditioners often do dangerous jobs and medical evacuations, when possible, are extremely expensive and logistically complex [10]. Thus, Antarctic winter expeditioners undergo rigorous physical and psychological testing to ensure they are optimally healthy and can live in close quarters for long periods of time with few people, including only one doctor and limited medical supplies.

There is a large multi-disciplinary body of literature over a period of 50 years discussing the range of psycho-social issues faced by polar expeditioners [11] and astronauts [12, 13] as well as those in other types of ICE environments (e.g., submarines) [14] and small group/team dynamics [15]. There is also a growing body of research focusing on the organisational culture of groups in ICE environments [16]. While a detailed examination of this literature is beyond the scope of this article, we provide highlights from the ICE environments literature below [7]. Isolation is the experience of physical separation from others and one’s typical social network. For example, expeditioners live and work in Antarctica for an extended period depending on their role (e.g., weeks, months, or years) and can experience varying levels of emotional and cognitive deprivation. Confinement is defined by restricted physical mobility due to the dangerous conditions in the working environment. For instance, expeditioners on vessels or on station live in confined quarters with limited physical space and privacy. There is little separation between work and leisure. During winter, it is difficult to leave the station to escape this confinement given the conditions. On a vessel, it is impossible to leave at any time. Thus, expeditioners and others working in ICE environments must emotionally cope in these situations and find ways to minimise their distress. The extreme environment itself poses a constant stressor despite improvements in living conditions on stations/vessels over time. This is a ‘wicked’ challenge that the organisation and individual need to manage.

The consequences of living with the psychological stressors associated with ICE environments are not limited to individuals: interdependent teams can also ‘catch’ each other’s emotions–referred to as emotional contagion [17]. With advances in communication technology and transportation as well as decreased exposure to occupational hazards, some of the physical and psycho-social issues that have been significant for people in ICE environments in the past are not necessarily as pressing today [18]. Although new challenges associated with being more able to communicate have emerged, expeditioners are now increasingly exposed to stressors over which they are unable to exert influence (e.g., COVID-19 pandemic). There is also a significant body of scholarship discussing the salutogenic effects of Antarctic employment, including a sense of personal achievement, increased self-esteem, resilience, and improved health [19]. This scholarship is important in understanding the appeal of undertaking multiple return expeditions, as well as providing hope for those in analogous environments in which a pathogenic paradigm continues to predominate.

### Organisational culture in Antarctica

Several studies have documented that organisational culture is a powerful force affecting an organisation’s well-being and effectiveness [20]. Most scholars agree that the key characteristics of ‘organisational culture’ include that it is holistic, ‘soft’ with respect to influencing behaviour by nurturing people to commit to their job, and difficult to change; has a historical basis; and is socially constructed [21]. Organisational culture is deeply held and shared [22]. Therefore, organisational culture serves as a frame of reference that allows members to make sense of their environment and their experiences and to share these experiences, feelings, and thoughts with others [23]. Organisational cultures typified by open communication strategies, perceived staff-focus, and trust lead to increased adaptive capacity and more resilient outcomes [24].

Culture is an important moderating factor in ICE environments in that it provides an organising framework for expeditioners to cope with the stressors of Antarctic life [25]. There is a small body of research focusing on Antarctic station culture [15, 26]. Notably, Antarctic stations and vessels have their own ‘microcultures’ that are defined via ‘…a process of negotiation between the needs of the individual and the needs of the social group’ [25]. Culture is a product of this negotiation and a set of rules that regulate this process [25]. In other words, adaptation to an Antarctic environment involves the interaction between the individual and the group [25]. In the first stage of adaptation, when expeditioners initially arrive in Antarctica, the group is comprised of people from different social backgrounds and values [25]. The social dynamics on station/on vessel are formed through open interaction and identification of shared common interests as well as differences and dislikes among the group members.

In the next stage, subgroups form based on shared interests or sociodemographic characteristics or leisure activities. This is not unlike processes observed in more routine environments. In some instances, these subgroups can become exclusive and lead to cliques which can threaten the success of the expedition and/or expeditioner well-being. Antarctic cultures that are characterised by cliques have higher levels of anger, anxiety, and depression [18]. This poses a threat to both individual and station-level performance. In the third stage, the entire group coalesces around a shared identity with group norms and behaviours. However, some people may be ostracised from the group for various reasons. Cultural issues on station can arise when ‘guests’ (e.g., new group members) join the existing group (‘hosts’) who are already adapted to station/vessel life [27]. Whilst the arrival of new expeditioners can alleviate social monotony, research from long-duration space missions shows that in playing the ‘host’, existing group members may feel less productive in having to support the ‘guests’ [28]. The ‘host-guest’ phenomenon can lead to psychic tension because new group members disrupt established routines and redistribute the station’s territory [27] which can lead to stereotyping and stigmatising of people who belong to different social groups [29].

Although station culture may change annually given the transient workforce, the literature shows that there is some continuity produced by the more stable features of station environments, such as material artefacts (e.g., mementos of Heroic Era expeditions) and rituals (e.g., midwinter polar plunges) that reinforce a culture of survival and adventure [25]. Group behavioural norms also underlie the culture, especially in relation to common spaces where expeditioners interact (e.g., gym, mess/eating areas). Expressions of these norms in mess/eating areas often include cleaning up after yourself and performing common duties (e.g., assisting the chef). These norms are governed within a broader set of norms around a set of personal qualities that define the typical expeditioner in that context (e.g., flexible, self-sufficient, etc.) as well as social identity (e.g., social class, gender, race/ethnicity, etc.) and status as summer or winter-over support [25].

The culture or social context is also an important factor in determining the development of an individual’s psychological contract with the organisation. Psychological contracts are defined as ‘the individual beliefs, shaped by the organization, regarding terms of an exchange agreement between individuals and their organization’ [30]. Individuals form beliefs about whether their psychological contracts have been fulfilled or breached based on their social environment, including their direct interactions with other employees. If an employee perceives that the organisation has fulfilled the psychological contract, the employee will have strengthened affective and emotional ties to the organisation [31]. Individuals are more likely to feel like their psychological contract has been breached when the organisation is performing poorly, when they have not experienced a formal process of socialisation, or when they have little interaction with other employees before they are hired [31]. Sarris [32] has demonstrated that there is a relationship between expeditioner perceptions of organisational culture (e.g., behavioural norms) and their job satisfaction. Thus, when expeditioners feel positively about the culture, they are more likely to recommend Antarctica as a good place to work.

COVID-19 has profoundly impacted organisational cultures globally [33]. Familiar symbols, rituals, and norms associated with organisational life have changed to accommodate reduced social contact and new hygiene practices. Whilst these measures have been necessary, they have also been extremely disruptive–especially social distancing. There is an emerging literature focused on the adverse mental health consequences associated with social isolation during the pandemic [34]. Social distancing practices send a psychological message to fear others as they might be disease carriers [35] and this fear can be precipitated by media exposure [36].

The disruption to social relations is arguably more acute in an ICE environment. Antarctic expeditioners are prepared to work in a challenging environment as part of their jobs. However, as Suedfeld and Steel [28] suggest, how people experience the environment is more important than the objective environment itself. Many expeditioners return to Antarctica multiple times despite the assorted stressors because of the positive affiliation they feel with the polar environment and the positive experience of social cohesion and camaraderie generated in sharing the experience with others [37]. The right balance of social interactions and physical proximity are important in an ICE environment as this helps expeditioners cope with the isolation and confinement [38]. Social restrictions can impact the development and consolidation of social relationships as well as the processing of experiences which can influence psychological adaptation and coping. Social restrictions may also make expeditioners more attuned to psychological contract breaches, and this can induce anxiety, depression, and/or emotional exhaustion [39].

The profound social dislocation introduced by the pandemic presents an important opportunity to study its impact on the organisational culture in Antarctica, including how COVID mitigation measures such as social distancing and quarantine reshaped the social experience of working in Antarctica.

### National Antarctic Program (NAP) responses to the COVID-19 pandemic

As soon as COVID-19 was first reported in Wuhan, China in December 2019, NAPs were planning how they would contain the virus, aware that an outbreak would have catastrophic consequences for Antarctic science and tourism, given the limited availability of medical interventions. In a normal summer season, around 4,000 people work in Antarctica. In addition, thousands of tourists visit each year–in 2019–20, totals reached nearly 74,000 people [40]. Because expeditioners can reach the continent more easily now, environmental and human control measures are critical.

To keep Antarctic COVID-19 free, most NAPs reduced operational capacity and ceased scientific work beyond keeping stations functioning [41, 42]. NAPs rapidly implemented pre-expedition quarantine and testing to protect the expeditioners who were already in Antarctica and transport new personnel to Antarctica safely [43]. There were also many changes in logistics, including shipping and aviation, which are the primary means of getting cargo and people to Antarctica [44]. NAPs also prepared medical resources and infrastructure to mitigate potential COVID-19 infections.

For most of 2020, the protocols implemented by NAPs were highly effective. However, Antarctica’s position as the planet’s last COVID-free zone ended on 22 Dec 2020, when 36 expeditioners tested positive for the virus at Chile’s Bernardo O’Higgins station [45]. A second station on King George Island in the South Shetlands also reported an incidence. The outbreak eventually totalled 58 cases, including both military and civilian personnel. COVID-19 outbreaks occurred again in December 2021 with 11 cases at Belgium’s Princess Elisabeth Station [46] and 24 cases at Argentina’s La Esperanza Station in January 2022 [47].

### Australian Antarctic Division and the Australian Antarctic Program

The Australian Antarctic Division (AAD) is part of the Australian Government’s Department of Agriculture, Water and the Environment. Based in Tasmania, the AAD leads and delivers the Australian Antarctic Program (AAP). The AAD workforce is split between the head office in Tasmania and its stations and in Antarctica. Expeditioners are people who work in Antarctica in a variety of roles. All expeditioners are trained and equipped at the AAD. There are approximately 300 staff at the AAD head office undertaking operational, medical, science, policy, and support functions. Australia maintains three year-round research stations—Casey, Davis, and Mawson—and one on sub-Antarctic Macquarie Island. Each station is like a small town, featuring station leaders, tradespeople, scientists, doctors, chefs, and communications experts. There are also people supporting shipping and aviation activities. In a typical season, the population at each station ranges between 40 and 100 expeditioners over summer and 15 to 25 over the winter months. 500 expeditioners usually travel south with the AAP. During the pandemic, the AAP sent half as many expeditioners to keep the stations operational.

### COVID-19 preventative measures in the AAP

The pandemic forced substantial changes in AAP operations [44]. In 2020, the AAP focused on resupplying stations and changing over teams. To prevent COVID-19 from spreading on stations/vessels, the AAP implemented 14-day pre-expedition hotel quarantine in Hobart, Tasmania with COVID-19 PCR testing on days 3, 7 and 13 [48]. During quarantine, expeditioners undertake pre-expedition training online.

The AAP implemented a COVID-19 safety management system for stations and vessels using a traffic light colour system [48]. ‘Green’ refers to normal operations (no restrictions). ‘Amber’ applies to the first 14 days after departure from Hobart. In this condition, expeditioners undertake a14-day period of enhanced social distancing (ESD). For most expeditioners, the majority of the first 14 days following departure are served on a vessel. In ESD, everyone on the ship undergoes a daily health check (e.g., temperature check, identification of COVID symptoms), wears face masks in all indoor spaces, and remains 1.5 metres apart where possible. Meal services are staggered, and enhanced cleaning of common surfaces is undertaken at least twice per day. Common areas (e.g., bar, gym, etc.) are closed, and social gatherings are prohibited. No visitors are allowed in other expeditioners’ cabins/rooms. This is a marked departure from the highly social/interactive nature of voyages under normal circumstances–voyages are typified by regular formal and informal gatherings between expeditioners and vessel staff. In general, all expeditioners are requested to disinfect their individual work areas and avoid non-essential contact. When the ‘Amber’ 14-day ESD period is over and no COVID-19 symptoms are identified, the vessel can return to normal operations (Green). Many of the expeditioners on resupply voyages in 2021 were at sea for 100+ days and endured multiple periods of quarantine and ESD compared to those who travelled directly to station.

Expeditioners who fly to station or who arrive via vessel before the first 14-day period expires must also undertake a period of ESD using the Amber protocol. However, this period may vary depending on flight delays or amount of time at sea. In this case, newly arrived expeditioners on station follow a similar protocol as described above; however, they are segregated from the existing station community until they complete their 14-day ESD period. The existing station community cannot physically interact with new arrivals. This restriction also impacts on social integration and dynamics as well as the psychological adaptation of the individual expeditioners.

In the event of a suspected or confirmed COVID case, the station or vessel follows the ‘Red’ protocol. Any individual with COVID-19 symptoms must don a mask, isolate in their room, and contact the medical practitioner for testing. If they test negative, they must self-isolate until symptoms resolve. If they test positive, they must go to an isolation ward for treatment and potential air evacuation to Hobart. The Red protocol requires the station/vessel to lock down–all expeditioners wear face masks (not just new arrivals), all non-essential staff isolate in their cabins/rooms, and all common areas are closed. All meals are delivered to individual rooms or picked up by appointment. In Red, all common areas are cleaned immediately and four times per day thereafter.

The unfamiliar demands of ESD increase the cognitive load experienced by expeditioners. Cognitive load refers the level of demand placed on an individual’s mental resources (particularly working memory) at any one time [49]. This can negatively impact decision making processes and affect regulation, particularly under time pressure or ambiguous, which in turn may further impact individual and intrapersonal wellbeing, and social cohesion.

## Method

Our multi-disciplinary research group (sociology, psychology, cultural studies) initially came together through an international project aimed at understanding the impact of COVID-19 in Antarctica. Around the same time, Author 1 started a commissioned study examining individual attitudes and expectations of AAD organisational culture. Although COVID-19 was not the primary focus of this study, the pandemic significantly affected the everyday working practices of expeditioners and, therefore, their experiences of station culture. Key research questions included:

1. What are the attitudes and experiences of AAD employees?
2. What are AAD employees’ perceptions of organisational leadership?
3. How can the organisational culture of the AAD be improved?

Recruitment occurred indirectly by means of a general email approved by the Director of the AAD and Chief Scientist and sent to all employees in Tasmania and Antarctica. Participants self-selected into the study by contacting Author 1 directly to protect confidentiality of participation. Sixty-three staff volunteered to participate. Author 1 used a sampling matrix to purposively select participants based on occupational role, employment status/career point, features of social identity (e.g., race, gender, sexuality, age) and geographic location (Tasmania or Antarctica). Those who agreed to participate submitted consent forms.

Employer-based recruitment offers many benefits but can raise ethical issues. To address these, participants were assured that this research was being independently conducted and all informed consent documents indicated that participation would have no bearing on their employment or benefits. AAD supervisors did not directly recruit participants and were discouraged from discussing the research with potential participants. Participants were assured that participation was confidential and that the AAD would not have access to raw/identifiable data. This is particularly important in small, closed communities such as Antarctica and accompanying risks associated with not being able to return in a future season.

Author 1 conducted one semi-structured interview of up to two hours with each participant (n = 22 interviews) online or by phone between March and May 2021. Participants comprised AAD head office staff (n = 13) in Tasmania, expeditioners currently in Antarctica at study commencement (n = 5) as well as recently returned expeditioners (within 6 months of study commencement) (n = 4). Head office staff included people working in every branch of the organisation (e.g., Science; Assets and Infrastructure; Technology and Innovation; Policy and International; Antarctic Operations and Safety; and Strategy and Communications). Expeditioners comprised all positions represented in AAP expeditions including station/voyage leaders, medical practitioners, field training officers, cooks/chefs, technicians, tradespeople, and communications personnel.

Author 1 used an interview guide with open-ended questions drawn from themes in the relevant literature [32]. Participants were asked questions about themselves (e.g., age, gender, race/ethnicity, postcode, education, income, employment) as well as questions focusing on how they perceive the AAD’s organisational culture in Tasmania and/or Antarctica; their perceptions of AAD leadership; their suggestions for how to improve the organisational culture; as well as how COVID-19 had impacted their organisational experiences. The interviews provided answers to key questions including: how has COVID-19 affected the everyday working practices of expeditioners? The social dynamics on station? What are the realities of negotiating COVID-19 hygiene and social distancing protocols on station and on vessels? This article focuses exclusively on the data collected from expeditioners in relation to their experiences of the AAP in the context of COVID-19. All interviews were recorded with consent and transcribed verbatim. This study was approved by our University’s Human Research Ethics Committee.

Interview analysis was based on grounded theory–a qualitative methodology that emphasises a systematic inductive approach to data collection and analysis focusing on building theory from data rather than hypotheses [50]. Grounded theory was chosen because its inductive principles align with the exploratory aims of this research, allowing us to generate new theories about the experiences of expeditioners during COVID, where no previous research exists. Following the grounded theory method, data were analysed first by Author 1 by open coding, or surface reading transcripts, taking note of any striking words, phrases, or themes arising from the data. Authors 2 and 3 provided critical feedback on the initial interpretation of the data.

**Fig 1:**
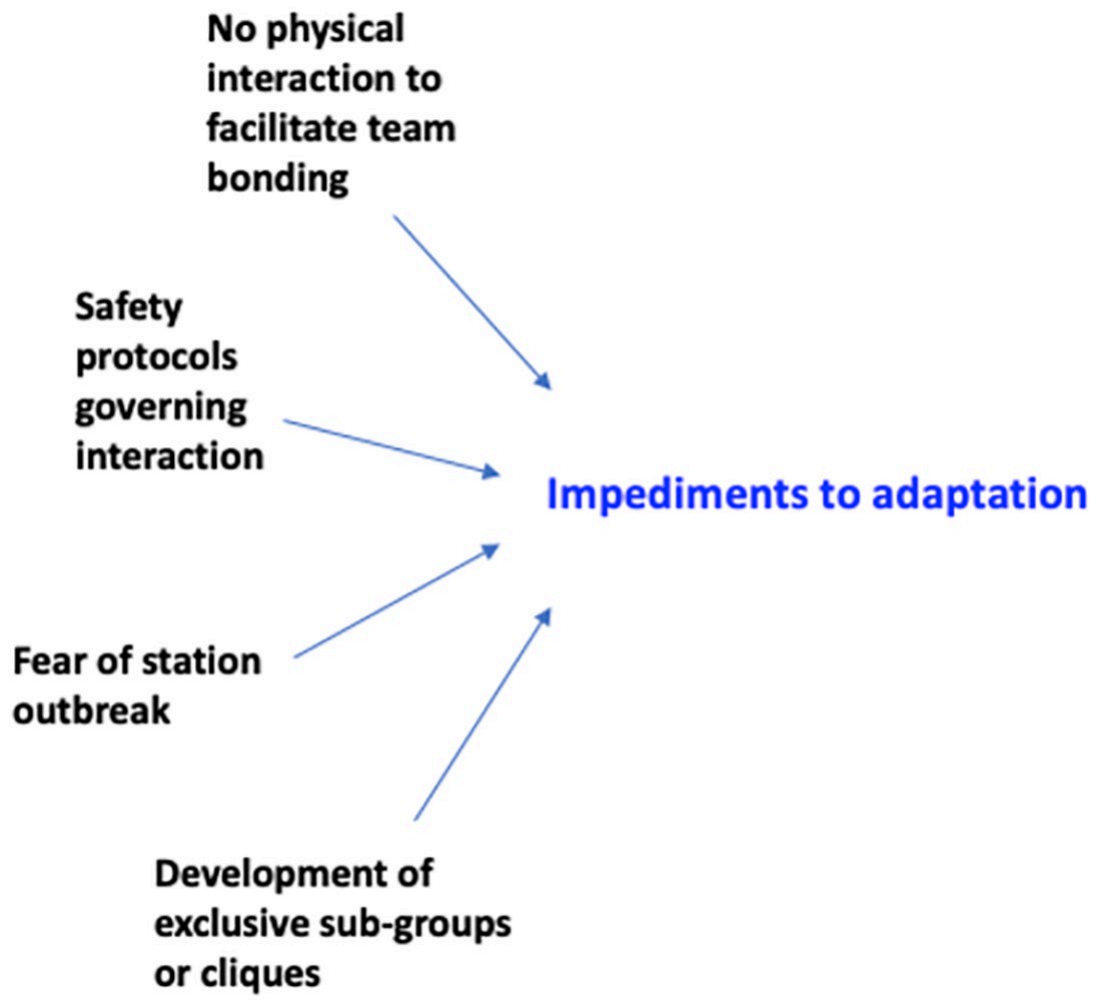
Illustration of how analytical categories were generated.

The main category of analysis was ‘impediments to adaptation’ and three key themes around this included learning about COVID in Antarctica; individual experiences of quarantine and enhanced social distancing; and the impact of quarantine and ESD on social interactions on station/on vessels. Fig 1 shows how the ‘impediments to adaptation’ category was generated from interview data. Theoretical coding or theory building was the last step of data analysis in which the research team looked for relationships between categories and themes.

Data have been de-identified, and pseudonyms are used throughout. The community of AAD employees in Australia is very small. We have provided no individual identifying information in the quoted extracts to protect the confidentiality of the participants. Participants in this study are aged between 30 and 66 years, with a mean age of 46 years. They come from 7 different countries mainly in the Anglosphere, with the majority (68%) born in Australia. Most expeditioners worked on fixed-term contracts given the seasonal nature of the work. All participants were positioned occupationally as middle class based on their yearly household incomes (on average AUD$125,000-$150,000). More than half (60%) of participants had a postgraduate degree and 86% of participants were white. De-identified data are available from the Australian Antarctic Data Centre. Formal requests for access to this data set require relevant justification and approval from the AAD Chief Scientist and/or AAD Director in line with AAD confidentiality and data security standards.

## Results

We discuss three key themes derived from responses to our research questions and in relation to participant’s experiences of COVID-19: 1) learning about COVID-19 in Antarctica; 2) individual experiences of quarantine and enhanced social distancing (ESD); and 3) the impact of quarantine and ESD on social interactions on station/on vessels. These themes cohere under the broader category of COVID-19 related adaptation challenges and discussed at length in forthcoming sections.

### Learning about COVID-19 in Antarctica

AAP expeditioners who were already in Antarctica in 2020 when the pandemic was unfolding had mixed experiences of learning about COVID-19.

I think it was January [2020] when we first heard about COVID, and first we thought that it’s just going to be like the SARs thing back in 2003 or 2004, and it’s going to be gone quick. By March we realised that the entire world is in lockdown. Then all those Hollywood movies come flooding in my head… I started to read about the virus, and how it spreads, and how it’s asymptomatic–and that makes it even worse. But even then, I didn’t realise that it’s going to go on for a year, and even more… Most of the time we felt lucky that we were in the station, safe and away from everything else…

(Participant 2)

It was like we were watching the whole world, whereas it’s usually the other way around. You feel a bit like a goldfish bowl and people [say], “How are you doing down there?”…[Some expeditioners] had family [overseas]…and that was a terrible worry for [them]. [They] had good social media contact with them though, so that was great…

(Participant 4)

In these extracts, participants positively described their general detachment from the fear, anxiety, and social distancing practices happening elsewhere. These extracts reflect participants’ feelings of detachment as reported with Australian (and other) media discourse, but equally interesting is that access to the media–mainstream and social–also *produced* this effect. For instance, Participant 2 notes that ‘Hollywood movies’ came ‘flooding’ into their head, provided a starting point for their understanding of the pandemic. This comment links to schema or the psychological constructs that guide expectations and engagement with new environments and contexts. In the absence of information, people tend to ‘fill in the blanks’ based on previous experience. Expeditioners had no first-hand experience of the pandemic so their understanding of COVID-19 was anchored in what they could watch or read online.

Although some expeditioners felt safe in their physical detachment from COVID-19, in line with some of the mainstream media articles, they were not immune to the negative emotions conjured by the pandemic. Participants in this study contended with a generalised fear that an outbreak could occur on station.

We were affected the most in the first two months [of 2020] because we were still [learning] to deal with this [pandemic]. You are sneezing. You have a dry throat. You just worry about it becoming a sore throat and what if it’s the real deal and you’re not reporting soon enough [to the doctor] and you’re just hurting the whole community.

(Participant 11)

This extract reflects the psychological stress felt by expeditioners. The increased reliance on the media for information about the pandemic and the high personal relevance of the threat of social rejection given the ICE environment would predictably increase expeditioners’ levels of fear [36].

### Individual experiences of hotel quarantine and enhanced social distancing (ESD)

In 2020 and 2021, AAP expeditioners undertook hotel quarantine in Hobart for 14 days before departing for Antarctica. Quarantine was challenging for participants in terms of managing isolation and lack of bonding with other expeditioners.

[Quarantine] makes [work] more tiring. In some ways it makes it easier. I really like having peace and quiet when I’m trying to think and planning things…I thought it was quite helpful. But the isolation is painful for a lot of people, and they struggle.

(Participant 19)

Periods of quarantine and ESD were significant for participants on resupply voyages given the duration of the voyages (between 30–100 days) and the multiple groups of people moving on and off the vessels.

We spent two weeks [in hotel quarantine in Hobart], but then we did ESD on the ship [multiple times]. And then we did a voyage…So the period when you can’t interact with anyone other than your direct work colleagues is a significant amount of time. It’s more isolating being on a ship…than it is being on station. It’s quite different experiences.

(Participant 19)

Psychological distress was also compounded for expeditioners because they had little control over their environments due to changing COVID-19 alert levels, an example of the negative impact that intolerance of uncertainty and increased cognitive load associated with a lack of unpredictability and control can have on psychological health and wellbeing.

…it’s just having some sense of control… It’s [finding] something for people to do and get their brains on and it’s positive…just providing people the basics, somewhere to gather, there was none of that

(Participant 22)

Although expeditioners are selected for their high adaptive capacity, as Participant 22 observes above, multiple periods of confinement and ESD were isolating and presented an additional mental burden. A perceived lack of control can not only impact individuals but also indirectly impacts the social cohesion of the entire community through emotional contagion and rumours. Significant and ongoing experiences of reduced social interaction may have increased expeditioners’ perceptions of psychological contract breaches.

### The effect of quarantine and ESD on station and vessel culture

Participants described how quarantine and ESD measures influenced the bonding between expeditioners and the establishment of communities.

I was really looking forward to that time in Hobart where you get to meet people… you get to share in the excitement building up towards the season and going down South and it all kicks off. That was dampened quite a lot by the quarantine. Then, coming on to station and then we had two extra weeks of [enhanced social] distancing …

(Participant 14)

…It would’ve been nice to be able to catch up with people, start forming those relationships before we went down [to Antarctica]. [Quarantine] caused a staggered arrival on station… So cliques formed based on which flight and which quarantine group you went through with…

(Participant 3)

[Hotel quarantine] was easier than the two weeks in ESD on station, which some people might think is strange, but when you’re in a room and you know that you can’t see anyone …on station you could see people, you could interact with them in the workplace with a mask on but you had to eat separately to them… For me [the hardest part] was knowing people on station. Couldn’t have that interaction…

(Participant 20)

As Participants 3 and 14 observe, inherent in the pre-departure time in Hobart is the shared experience of excitement about the Antarctic expedition. The lack of physical time together makes setting up the station/vessel organisational culture difficult. Participant 20 makes an interesting observation around the psychosocial impact of quarantine versus ESD. They note that quarantine was easier perhaps because they were psychologically prepared to avoid physical interaction with anyone for two weeks. In comparison, ESD contravenes what expeditioners enjoy about being on station and they were perhaps not as well prepared for it during pre-departure. Participant 20 found it painful to be amid so many opportunities for social connection on station without being able to partake in them.

The delayed development of a microculture on station stifled the development of social relationships between new and existing expeditioner groups.

[Because of the staggered arrival of expeditioners] they form their little groups and when you’re later in the season…the station is established, it does have its groups and it is hard to find your way in…Sometimes you do feel like you’re on the outer…that was more noticeable this year with these COVID bubbles… On station there was a clear delineation of ‘we’ve been on station longer, we don’t wear face masks anymore… you don’t eat in the same area as us, you eat in a separate area’.

(Participant 20)

…When we got to station, we weren’t allowed to have the traditional handover…it was very strange not to be able to go up and hug these guys and give them the big embrace and a handshake and catch up. We had to keep [in ESD], and that was very weird…

(Participant 12)

The extracts above reflect the processes that are often observed between incoming and outgoing teams of expeditioners on stations. The incoming team members are seen as ‘outsiders’ and there is limited social interaction with them. The incoming members often do not feel like they belong until the outgoing team leave the station. The key difference here is that during COVID-19, this is an ongoing experience for expeditioners as incoming team members are trying to engage with an existing team.

The AAP continually revised its COVID protocols as the evidence base grew to ensure they were fit for purpose. However, this was challenging for the expeditioners because the rules for behaviour were shifting frequently. This caused uncertainty and change fatigue.

…Different rules on different days wore people down…You never knew what you were going to get when you got up in the morning. …It’s just that uncertainty that eats away at people. When you’re already going to a place that’s frontier and you’ve got a huge amount of uncertainty. I’ll say I’m good at it to a level and then I’m not. It just becomes too much.

(Participant 22)

Multiple layers of uncertainty related to COVID-19 protocols made it hard for expeditioners to plan and feel as though they were in control of their environment and could rely on their community for support. Workplace disruptions also perhaps increased expeditioners’ awareness of a psychological contract breach.

## Discussion

At the start of this article, we argued that the media provided a romanticised and limited view of Antarctic expeditioner experiences during the pandemic by focusing on the paradoxical combination of their ideal modelling of survival in isolation and their location in the ‘safest’ place on Earth. In contrast, this study shows that expeditioners grappled with many of the same problems as the global isolated 2020 workforce–they managed new work contexts, had minimal preparation to build teams and socialise in a digital environment, negotiated social distancing, and felt the uncertainty that comes with changing hygiene protocols and not having a set expedition end-date.

Drawing on expeditioner narratives and conceptualised through grounded theory methodology, our findings reinforce the idea that life in Antarctica can provide important insights for managing survival in a pandemic. However, these insights rest not on individual heroism but on psychological and social dynamics. While surviving alone, in the manner of Richard Byrd, provides one kind of challenge, surviving in close quarters with other people provides another. This study reinforces much of the adaptation literature in terms of the importance of contemporary Antarctic communities as important social support resources for mitigating the psychosocial effects of a global pandemic. The existing literature shows that the social environment strongly shapes how expeditioners perceive the organisation [26]. Organizational culture also plays a significant role in the creation of expeditioners’ perception of psychological contract fulfilment. However, this study builds on and expands the adaptation literature by highlighting how the pandemic amplified the importance of individual expeditioners in coping and adapting to the environment versus the community.

**Fig 2:**
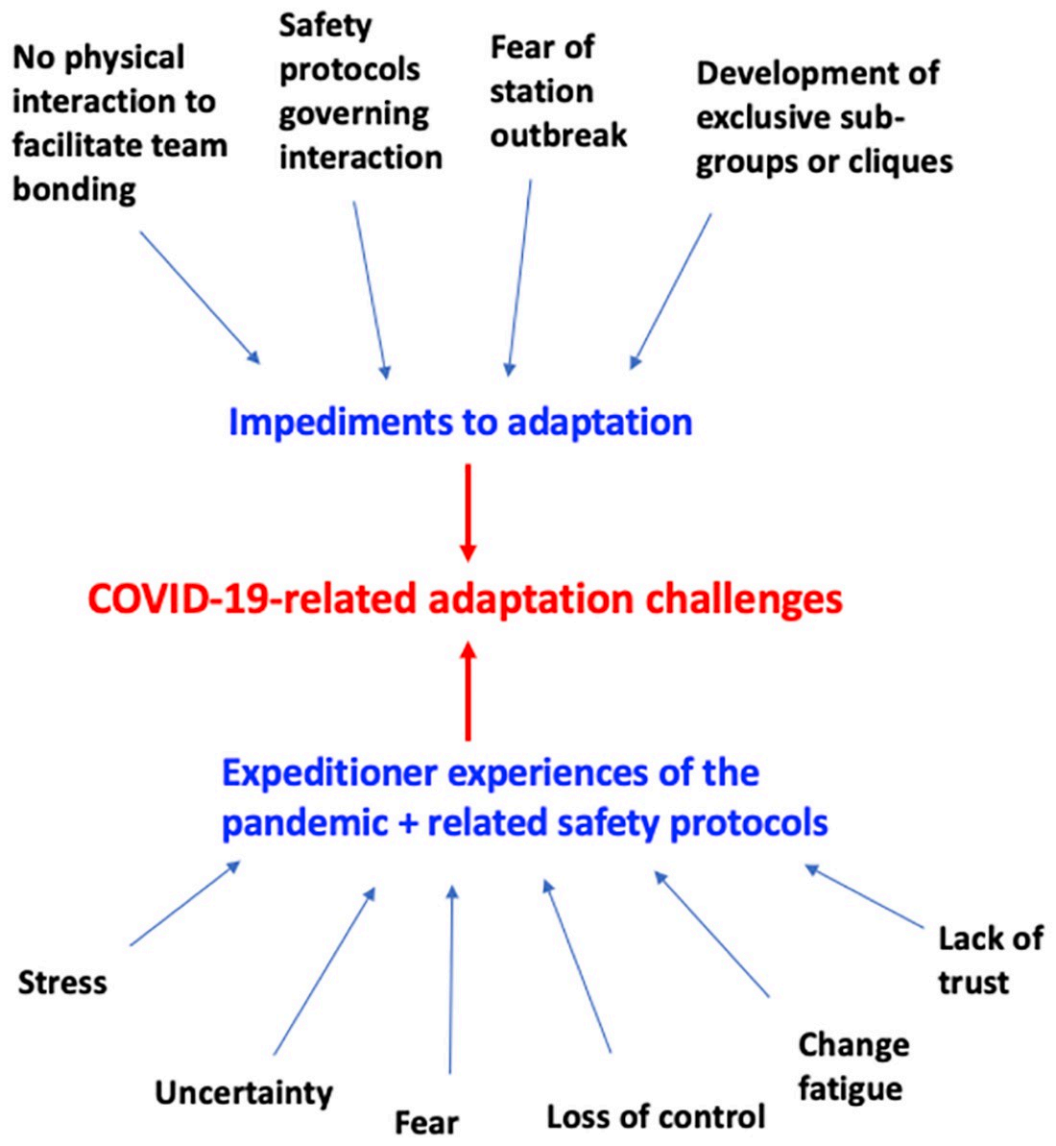
Conceptual model describing COVID-19 related adaptation challenges for expeditioners.

In this context, the issue of group adaptation was acute during the pandemic. As opposed to allowing groups to form organically, the pandemic required that the AAP develop rules that governed expeditioners’ interactions with one another, undermining the first stage of adaptation. This is a critical state that helps the group to cohere around share values upon arrival in Antarctica. Whereas status inequality in the group may have developed from other features of social identity (e.g., job role) in a typical season, during the pandemic, status inequality was predicated on which COVID-19 quarantine group you arrived in, whether you were wearing a mask or not, and/or how long you had been on station. As a result, in the second stage of adaptation, exclusive subgroups or cliques formed in response to the unusual social environment (e.g., expeditioners socialised with their others in their COVID ‘bubbles’) [18]. This is different to typical seasons in which such cliques are primarily based on occupational role. In this study, expeditioners had great difficulty in achieving the third stage of adaptation around a shared identity and group norms as new arrivals to station were not able to be incorporated into the collective in the normal ways [28]. These findings are outlined in our conceptual model where COVID-19 related adaptation challenges sit at the centre of the model (Fig 2).

Our study shows that COVID-19 profoundly impacted the organisational culture of stations. The consequences of social dislocation, however, are amplified in extreme communities compared to other workplaces globally as this is layered on top of geographic dislocation and the associated dislocation from family and friends. This situation is high risk for NAPs because this it can undermine trust in the organisation if expeditioners feel like it is solely up to them to cope during a pandemic. Expeditioners in this situation are more likely to perceive psychological contract breaches and are at increased risk of exceeding cognitive load, decreasing cognitive flexibility, and increasing risk of burnout which can impair the completion of an expedition [39]. Moreover, expeditioners may be less likely to want to return to Antarctica for a subsequent season if they have had a negative experience [32].

## Conclusion

Although global pandemics are not in an organisation’s control, our findings have clear policy implications for NAPs. We have shown that health interventions for COVID-19 cannot be disconnected from the psychological and social forces that are present at a local level (e.g., on station or vessel). Moreover, stable and vibrant Antarctic communities are critical sites for managing programs during a pandemic–NAPs cannot underestimate the importance of social relationships in a pandemic. Investing intentionally in organisational culture and interpersonal relationships can assist NAPs to retain levels of interconnectedness and well-being despite the requirement that expeditioners quarantine and socially distance during COVID-19 for the foreseeable future. This, in turn, can increase expeditioner retention and productivity. For example, findings demonstrate that pre-departure hotel quarantine is perhaps a critical time setting expectations and the ‘microculture’ of the group. This is especially important given that expeditioners are working with people not of their own choosing–expeditioners are selected as individuals rather than groups.

Moreover, in times of crisis, when an environmental trigger (like a pandemic) is more than the organisational culture can absorb, it is incumbent upon leaders (e.g., station leaders) to observe these changes and monitor fluctuations in day-to-day events on station and re-focus the group on the mission and values of the organisation. It is also important that NAPs invest more intentionally in virtual/digital tools (e.g., harnessing pre-departure training online) to ensure that the team is sufficiently bonded and aligned around the mission before the expedition.

COVID-19 will be with us for a long time in different forms, and no doubt Antarctic expeditioners (along with astronauts) will continue to be popularly understood and portrayed through the lens of heroic endurance. Qualitative, interdisciplinary studies of this kind will be increasingly important to understanding the social and personal complexities that lie beyond familiar stereotypes of expeditioners. To date, much of the relevant scholarly attention in the adaptation literature has focused on the individual health and well-being of Antarctic expeditioners. Our findings speak to the importance of bringing together multiple bodies of literature including the insights from polar psychology and sociology and the relevance of broader individual, socio-cultural, and historical issues related to the impact of COVID-19 on expeditioners. The latter includes how expeditioners cope individually and interact in the context of COVID protocols, the factors that influence the coherence of teams/community, and ultimately, how these factors together affect the outcome of the expedition. Future Antarctic expedition teams will be required to work effectively under complex and dangerous conditions to successfully accomplish their missions.

Through an interdisciplinary understanding of individual and team dynamics in a pandemic environment, we can minimize potential threats to expedition success while optimizing team performance. Importantly, this exploration of interpersonal relationships in a polar environment during COVID-19 can inform the types of interactions that may occur during short and long duration space missions. As COVID-19 variants become more common and protocols advance, it is essential to continue to extrapolate information from analogue groups in Antarctica to provide an evidence base to inform policies and practices that consider the interrelated factors influencing human health and performance in such contexts and more broadly in non-ICE environments.
